# The Motoric Types of Delirium and Estimated Blood Loss during Perioperative Period in Orthopedic Elderly Patients

**DOI:** 10.1155/2018/9812041

**Published:** 2018-11-01

**Authors:** Narei Hong, Jae-Yong Park

**Affiliations:** ^1^Department of Psychiatry, Hallym University Sacred Heart Hospital, College of Medicine, Hallym University, Anyang-si, Republic of Korea; ^2^Department of Orthopaedic Surgery, Hallym University Sacred Heart Hospital, College of Medicine, Hallym University, Anyang-si, Republic of Korea

## Abstract

**Background:**

Delirium is a common and serious syndrome in elderly patients. The hypoactive type of delirium is known to have different characteristics, but further studies are needed to define the specificities of these characteristics. Our study aims at finding specific risk factors, especially estimated blood loss during operations of hyper- and hypoactive delirium in orthopedic elderly patients.

**Methods:**

One hundred and seventy-five elderly patients were evaluated using the Confusion Assessment Method (CAM) and the 4^th^ edition text revision of the Diagnostic and Statistical Manual of Mental Disorders (DSM-IV-TR). Trained psychiatrists interviewed the subjects directly at pre- and postoperative time points. We reviewed medical records after the patients were discharged.

**Results:**

Thirty-nine patients (22.3%) were diagnosed with multiple types of delirium, which included 17 hyperactive types (65.9%), 13 hypoactive types (33.3%), and 9 mixed types (23.1%). Although the mean estimated blood loss in patients with either hyper- or hypoactive symptoms was larger than in patients lacking these symptoms, the odds ratio was only significant in patients with hyperactive symptoms. In addition, age, preoperative daily function, and preoperative hyponatremia were found to be risk factors for hyperactive but not hypoactive symptoms.

**Conclusion:**

Patients with hypoactive symptoms had different risk factors than patients with hyperactive symptoms of delirium. The estimated blood loss, well-known risk factors for delirium, might be risk factors for only hyperactive delirium. The acute precipitating factors seemed to show stronger correlation with the hyperactive type of delirium than with the hypoactive type.

## 1. Introduction

Delirium is a common and serious syndrome in elderly patients. It is one of the most cost consuming problems in the medical systems of aged societies. More specifically, postoperative delirium is a common and disturbing complication for surgeons [1–4].

The Diagnostic and Statistical Manual of Mental Disorders, 5^th^ edition (DSM-5), defines delirium as a disturbance of attention or awareness that is accompanied by a change in baseline cognition that cannot be better explained by a preexisting or evolving neurocognitive disorder. It has two kinds of specifiers: persistency (acute or persistent) and the level of activity (hyperactive, hypoactive, or mixed)[5].

The prevalence of postoperative delirium varies from 15 to 53% in older individuals depending on the settings and populations; it is in any case too high to be neglected. In addition to the high prevalence, the prognosis of delirium is very poor. Although the majority of individuals with delirium recover fully, the disease can give poorer consequences including impaired postoperative recovery, increased risk of nursing home placement, prolonged hospitalization, and increased mortality. [4–6].

Although delirium tends to be underdiagnosed, early recognition and intervention can prevent the development and progress of the disease[7–9]. Numerous studies exploring the risk factors of delirium have separated them into two categories: the predisposing factors (age, cognitive symptoms, psychiatric disorders, medical comorbidities, and premorbid functions) and the precipitating factors (medication, physiological disturbances such as electrolyte imbalances and metabolic acidosis, dehydration, fractures, hypoxia, infection, pain, catheterization, and environmental change)[3, 4, 10–12]. Intraoperative factors such as large blood loss during operation, long operation time, and high volume of transfusion can also precipitate delirium.

Blood loss may be one of the most important intraoperative and also perioperative precipitating factors in elderly patients who undergo surgical procedures. As the amount of blood loss cannot be measured directly during operation, many formulae strive to calculate the accurate amount of blood loss by estimating the volume of red blood cells or the volume of whole blood using either the hematocrits or the hemoglobin of patients[13].

Besides hyperactive delirium which is commonly known in clinical situation, nowadays hypoactive delirium has started to attract attention from many clinicians and researchers. Although the symptoms of hypoactive delirium are silent, recent studies have recognized prognostic significance of hypoactive delirium[14–16]. Avelino-Silva et al. further reported that patients with hypoactive symptoms have worse prognosis than patients from other types of delirium[17]. Also because of the characteristics of symptoms, many hypoactive patients do not get the attention they need and there are not enough studies on hypoactive delirium yet. So specific characteristics of hypoactive delirium are still unclear[15].

In this study we aim at unveiling the specific risk factors of hyperactive and hypoactive delirium. Here we hypothesize that hyperactive and hypoactive types of the disease might be differently affected by blood loss during operations. We further hypothesize that elderly patients who underwent orthopedic surgery and had large blood loss during operations were at bigger risk of developing delirium.

## 2. Materials and Methods

### 2.1. Study Population

One hundred and seventy-five elderly patients who were admitted at the Department of Orthopedic Surgery, Hallym University Sacred Heart Hospital, between May 2013 and May 2014 were enrolled in this study before they underwent surgery. They were all over 65 years old at the time of admission. The surgery was about the bone treatment from fracture fixation to artificial joint. The patients with simple procedures like hardware removals were excluded because only the patients admitted more than five days were enrolled for estimation of the perioperative blood loss. We excluded the subjects who could not communicate verbally as well as those who could not take the evaluation process due to serious medical problems or brain injuries at the time of admission.

All patients gave their informed consent and the Institutional Review Board of the Hallym University Sacred Heart Hospital approved this study.

### 2.2. Data Collection

The subjects were evaluated three times: on the first or second hospital day, on the second postoperative day, and after discharge. The first two evaluations were directly done by psychiatrists whereas we reviewed the medical chart for the third evaluation after the patients' discharge.

At admission, demographic data, baseline functional disability, diagnosis of dementia, and medical history were confirmed by the patients and their primary caretakers. Pre-, intra-, and postoperative parameters were obtained by reviewing the medical records.

### 2.3. Delirium Assessment

The Confusion Assessment Method (CAM)[18, 19]. was used to evaluate the patients for delirium at the first and second evaluations. CAM, an instrument with high sensitivity and specificity, is the most widely used method of delirium detection in clinical setting[18, 20].

To diagnose delirium and dementia clinically, psychiatrists interviewed the patients using the Diagnostic and Statistical Manual of Mental Disorders, 4^th^ ed., text revision (DSM-IV-TR)[21]. As enrollment had started in May 2013, we could not use the DSM-5.

As there are no subtype specifiers for delirium in the DSM-IV-TR,[21]. we referred to the Assessment and Treatment of Delirium of Canadian Coalition for Senior's Mental Health to classify the different subtypes (depending on the level of activity)[22, 23].

All the assessments were done by trained psychiatrists through face-to-face interviews.

### 2.4. Estimating the Amount of Blood Loss

We used Mercuriali's formula to estimate the amount of perioperative blood loss. Mercuriali's formula estimates the volume of red blood cells (RBC) based on the preoperative hematocrit (Hct_preop_) and the fifth postoperative day hematocrit (Hct_day5  postop_). This formula is suitable for estimating blood loss in surgery in clinical setting as it has the advantage of being very clear in time and user friendly[13, 24].

  Estimated  blood  loss = blood  volume × (Hct_preop_ − Hct_day5  postop_) + ml of transfused RBC

The blood volume was estimated using Nadler's formula[25].

  Men: 604 + 0.0003668 × [size(cm)]^3^ + 32.2 × weight(kg)
  Women: 183 + 0.000356 × [size(cm)]^3^ + 33 × weight(kg)

### 2.5. Statistical Analysis

Statistical analyses were performed using both univariate (independent t-test and Pearson's chi-squared test) and multivariate (logistic regression) procedures. We classified the groups into delirium group and control group due to diagnosing process with DSM-IV-TR. We used Statistical Package for the Social Sciences Statistics 24 (SPSS 24) (IBM) for analyses and we set significance at P<0.05 (2-sided).

## 3. Results

We screened 532 patients and we enrolled 175 patients with informed consents (mean age of 74.06), 124 of which were women. 39 patients were diagnosed as delirium: 17 hyperactive types, 13 hypoactive types, and 9 mixed types.  Table 1 shows the demographic and perioperative characteristics of the subjects.

Subjects with delirium were older (p=0.001) and had lower educational level (p=0.006) than those without delirium. They did not have dementia more frequently (p=0.126) but they did show lower daily functioning compared to subjects without delirium(p=0.005).

We also found lower preoperative levels of hemoglobin (p=0.038), hematocrit (p=0.040), and sodium (p=0.008) as well as lower postoperative hemoglobin level (p=0.006) in the elderly with delirium compared to those without. Similarly, patients with delirium had higher preoperative and postoperative lactate dehydrogenase (preoperative, p=0.013; postoperative, p=0.042) and postoperative C-reactive protein (p=0.011).

The estimated mean volumes of blood loss during operations were larger in delirium patients (p<0.001). In multivariate stepwise logistic regression, the odds ratio of the estimated volumes of blood loss for delirium was 0.999 (p=0.002, 95% CI [confidence interval]=0.998-1.000). Patients who had hyperactive type (1053.17±839.19) and mixed type of delirium (1298.97±1485.26) lost larger amounts of blood during the operation than patients with hypoactive delirium (697.00±803.69) or without any types of delirium (521.52±599.85) (p=0.001).

The differences between patients with and patients without hyperactive symptoms of delirium and between patients with and patients without hypoactive symptoms of delirium showed different characteristics (see Table 2). Patients with hyperactive symptoms of delirium were older than those without (odds ratio; 0.917 (p=0.039, 95% CI=0.844-0.996)) whereas patients with hypoactive symptoms and patients without showed no significant difference in age. Patients with mixed activity felt more pain during the postoperative period compared to patients without mixed symptoms; however only the odds ratio of the pain for hypoactive symptoms was statistically significant (hypoactive symptoms, 0.691 (p=0.004, 95% CI=0.537-0.889); hyperactive symptoms, 0.768 (p=0.069, 95% CI=0.578-1.021)). The odds ratio of the estimated volumes of blood loss for hyperactive and hypoactive symptoms was 0.999 (p=0.001, 95% CI=0.998-1.000) and 0.999 (p=0.081, 95% CI=0.999-1.000).

For each symptom of delirium, patients with acute onset, inattention, altered consciousness, disorientation, perceptual disturbance, and altered circadian rhythm lost larger volumes of blood during the operation. More specifically, subjects with psychomotor agitations got larger estimated blood loss but no difference was observed in subjects with psychomotor retardation (see Table 3).

## 4. Discussion

Delirium is one of the most common postoperative syndromes in elderly patients. Efforts are being made by clinicians to diagnose delirium earlier and prevent or alleviate its poor consequences. The hypoactive symptoms of delirium remain however misdiagnosed [16] and insufficiently studied.

Here we study a group with 22.3% prevalence of delirium, among which 65.9% are of the hyperactive type, 33.3% of the hypoactive type, and 23.1% of the mixed type. Although previous studies showed a broad range of prevalence in each clinical setting, the prevalence and composition of subtypes used here are similar to previous studies[15–17].

Similar to other studies, patients with delirium were older and less educated and had poorer daily functioning than patients without delirium. No significant differences concerning dementia were observed between the two groups but this could be due to the low prevalence of dementia in our subjects as we had excluded from our study the subjects who could not communicate verbally and those who could not take the evaluation process due to serious medical problems or brain injuries at the time of admission, all of which are at high risk of having dementia.

Consistent with the known risk factors for delirium [4, 11, 26–28] we found electrolyte imbalance, the evidence of posttraumatic bleeding and tissue damage. Some studies suggest preoperative C-reactive protein as a risk factor for delirium [29] but C-reactive protein was only higher in patients with delirium at postoperative period.

Blood loss is one of the well-known risk factors for delirium [4, 11, 26–28]. However exact amount of blood loss in perioperative period cannot be measured in clinical situation. There are many attempts to estimate the amount of blood loss. Mercuriali's formula, which we used in this study, is one of the attempts, and it is known as the suitable formula for studies regarding blood loss in surgery [13].

Although estimated blood loss is widely studied on delirium, like other risk factors, there are scarce studies on estimated blood loss and motoric types of delirium.

A motoric subtype of delirium has been added to the last version of the DSM, the DSM-5. Despite the fact that only few studies focused on the hypoactive type of delirium, hypoactive delirium has specific characteristics and prognostic effects. Hypoactive delirium is more common in critically ill patients such as those in intensive care units or cardiosurgery units, and the prognosis of patients with hypoactive delirium seems poorer than that of hyperactive patients. Patients with hyperactive delirium seem to differ from hypoactive delirium patients both with their symptoms and with their prognosis. However, little is known about a potential difference between risk or etiological factors[15–17, 30]. To determine the risk factors for each motoric subtype, we compared the differences between patients with or without hyperactive symptoms as well as between patients with or without hypoactive symptoms. In our study, patients with hypoactive delirium had different risk factors than patients with hyperactive delirium. Age, poor preoperative daily functioning, and preoperative hyponatremia were risk factors for hyperactive but not hypoactive symptoms. Estimated blood loss during perioperative period was larger in patients with both symptoms than without. Mean postoperative potassium levels were lower in patients with hypoactive symptoms whereas postoperative hemoglobin and hematocrit which were influenced by perioperative bleeding were only lower in patients with hyperactive symptoms. Postoperative pain affected both types of delirium. Estimated blood loss during perioperative period was one of the most influential factors for delirium and both motoric subtypes. Although the means of estimated blood loss in patients with hyperactive symptoms and hypoactive symptoms were larger than for patients without hypo- or hyperactive symptoms, the odds ratio was only significant in hyperactive symptoms. To determine the influence of estimated blood loss in the perioperative period for each symptom, we performed independent sample t-tests for the subjects who had answered yes or no for every item of the CAM. The main symptoms of hyperactive type delirium had more difference in mean estimated volumes of blood loss during perioperative period. Patients with psychomotor retardation lost almost the same amount of blood as patients without retardation. Estimated blood loss during perioperative period thus seems to predict hyperactive but not hypoactive delirium.

In our study, the acute precipitating factors seemed to be more highly correlated with hyperactive delirium than with the hypoactive type. Although we could not find any specific difference in predisposing factors, hypoactive delirium might be more influenced by chronic predisposing factors than by acute precipitating factors, which could explain the poorer prognosis. There had been scarce studies on specific risk factors for hypoactive delirium. So there have been not enough studies to support our studies. Further investigations are needed to strengthen our hypothesis.

Many studies suggest that hypoactive delirious patients are neglected or misdiagnosed in the clinical setting as the symptoms of hypoactive delirium do not attract the clinician's attention. This also applies to our study in which many patients with hypoactive delirium did not have typical precipitating factors of delirium. As the prognosis of hypoactive delirium seems poorer than that of hyperactive delirium, clinicians should pay special attention to the hypoactive type.

The main strength of this study was to explore the risk factors for hyper- and hypoactive delirium separately. To diagnose the delirium and to classify motoric subtypes accurately, trained psychiatrists interviewed all the subjects face-to-face. More specifically we tried to understand the influence of estimated blood loss on delirium.

There were also some limitations in this study. First, the number of subjects was small as we prioritized the homogeneity of the group for this first study on estimated blood loss and motoric subtypes of delirium. Had the number of subjects been higher, we might had observed a cleared difference between hyperactive and hypoactive delirium. An additional study with a higher number of subjects and in different clinical setting would solidify our results. Second, there might be a selection bias in this study. The face-to-face interviews with psychiatrists seem to increase the accuracy of evaluation all while decreasing the consent rate. Third, we cannot interview subjects after long term follow-up meaning that the prognosis of both types of delirium cannot be analyzed in this study.

In this study, we suggest that risk factors for hyperactive and hypoactive delirium are different. While estimated blood loss volumes during perioperative period might affect hyperactive delirium, they do not seem to affect hypoactive delirium. The estimated blood loss, well-known risk factors for delirium, might be risk factors for only hyperactive delirium. Future studies should focus on hypoactive delirium.

## Figures and Tables

**Table 1:** Demographic and perioperative characteristics of the subjects.

	Delirium	Without Delirium	Total
	(N=39)	(N=136)	(N=175)
Age (years)_ _^*∗*^	76.95±6.48	73.24±5.77	74.06±6.12
Sex(male/female)	12/27	39/97	51/124
Education_ _^*∗*^			
None	17 (43.6%)	31 (22.8%)	48 (27.4%)
Elementary School	17 (43.6%)	50 (36.8%)	67 (38.3%)
Middle School	2 (5.1%)	26 (19.1%)	28 (16.0%)
High School	1 (2.6%)	25 (18.4%)	26 (14.9%)
Over College	2 (5.1%)	4 (2.9%)	6 (3.4%)
Dementia_ _^*∗∗*^	2 (5.1%)	3 (2.2%)	5 (2.9%)
Daily Function_ _^*∗*^			
Independent	21 (53.8%)	96 (70.6%)	117 (66.9%)
Need Help in Complex External Activities	9 (23.1%)	33 (24.3%)	42 (24.0%)
Need Help in Daily Living Intermittently	7 (17.9%)	4 (2.9%)	11 (6.3%)
Fully Dependent	2 (5.1%)	3 (2.2%)	5 (2.9%)
Estimated Blood Loss(ml)^ +,++^	991.17±1009.30	521.52±599.85	626.19±734.89
Red Blood Cell			
Preop^†^ Hemoglobin(g/l)_ _^*∗*^	12.02±2.15	12.66±1.52	12.52±1.70
Preop^†^ Hematocrit(%)_ _^*∗*^	35.75±6.00	37.54±4.35	37.14±4.80
Postop^††^ Hemoglobin(g/l)_ _^*∗*^	10.51±1.51	11.27±1.50	11.09±1.53
Postop^††^ Hematocrit(%)	31.04±4.42	33.28±4.29	32.77±4.41
Operation Time (min)	142.05±93.14	121.93±83.63	126.44±85.99
Anesthetic Time (min)	205.77±102.28	176.41±94.12	183.15±96.54
Electrolytes			
Preop^†^ Sodium (mmol/l)_ _^*∗*^	135.05±20.28	139.90±3.31	138.82±10.12
Preop^†^ Potassium(mmol/l)	4.21±0.59	4.29±0.52	4.27±0.54
Postop^††^ Sodium (mmol/l)	134.03±20.26	136.84±12.21	136.20±14.42
Postop^††^ Potassium (mmol/l)	3.65±0.40	3.88±0.49	3.83±0.48
Pain			
Preop^†^ Pain (VAS)	2.00±1.41	1.87±1.66	1.90±1.61
Postop^††^ Pain (VAS)_ _^*∗*^	1.59±2.02	0.75±1.35	0.94±1.56

^*∗*^P<0.05. ^*∗∗*^Diagnosed with Diagnostic and Statistical Manual of Mental Disorders, 4^th^ ed., text revision (DSM-IV-TR). ^+^The estimated volume of blood loss was determined through Mercuriali's formula.^++^P<0.001. ^†^The sample was taken on the first or second hospital day, before operation. ^††^The sample was taken on the second postoperative day

**Table 2:** The characteristics of the patients with hyperactive symptoms and hypoactive symptoms of delirium.

	Patients With Hyperactive Symptoms	Patients Without Hyperactive Symptoms	Patients With Hypoactive Symptoms	Patients Without Hypoactive Symptoms	Total (N=175)
	(N=26)	(N=149)	(N=22)	(N=153)	
Age (years)	77.31±6.24_ _^*∗*^	73.50±5.94	76.09±6.08	73.77±6.01	74.06±6.12
Sex(male/female)	9/17	42/107	5/17	46/107	51/124
Education					
None	9 (34.6%)	39 (26.2%)	11 (50.0%)	37 (24.2%)	48 (27.4%)
Elementary School	13 (50.0%)	54 (36.2%)	7 (31.8%)	60 (39.2%)	67 (38.3%)
Middle School	2 (7.7%)	26 (17.4%)	2 (79.1%)	26 (17.0%)	28 (16.0%)
High School	0 (0.0%)	26 (17.4%)	1 (04.5%)	25 (16.3%)	26 (14.9%)
Over College	2 (7.7%)	4 (2.7%)	1 (4.5%)	5 (3.3%)	6 (3.4%)
Dementia_ _^*∗∗*^	2 (7.7%)	3 (2.0%)	0 (0.0%)	5 (3.3%)	5 (2.9%)
Daily Function					
Independent	13 (50.0%)_ _^*∗*^	104 (69.8%)	13 (59.1%)	104 (68.0%)	117 (66.9%)
Need Help in Complex External Activities	6 (23.1%)	36 (24.2%)	4 (18.2%)	38 (24.82%)	42 (24.0%)
Need Help in Daily Living Intermittently	5 (19.2%)	6 (4.0%)	4 (18.2%)	7 (4.6%)	11 (6.3%)
Fully Dependent	2 (7.7%)	3 (2.0%)	1 (4.5%)	4 (2.6%)	5 (2.9%)
Estimated Blood Loss(ml) ^+^	1138.26±1082.06^++^	536.83±618.92	943.26±1140.72_ _^*∗*^	580.59±649.47	626.19±734.89
Red Blood Cell					
Preop^†^ Hemoglobin(g/l)	12.18±2.16	12.57±1.60	12.24±2.16	12.55±1.62	12.52±1.70
Preop^†^ Hematocrit(%)	36.18±6.10	37.31±4.54	36.44±5.95	37.24±4.63	37.14±4.80
Postop^††^ Hemoglobin(g/l)	10.37±1.51_ _^*∗*^	11.22±1.50	10.61±1.31	11.16±1.55	11.09±1.53
Postop^††^ Hematocrit(%)	30.45±4.44_ _^*∗*^	33.19±4.28	31.51±3.85	32.96±4.47	32.77±4.41
Operation Time (min)	136.35±100.35	124.79±83.37	146.59±97.22	123.52±84.20	126.44±85.99
Anesthetic Time (min)	201.15±116.39	179.90±92.61	209.55±96.62	179.22±96.23	183.15±96.54
Electrolytes					
Preop^†^ Sodium (mmol/l)	133.23±24.55_ _^*∗*^	139.79±3.48	138.41±4.34	138.88±10.70	138.82±10.12
Preop^†^ Potassium (mmol/l)	4.32±0.66	4.26±0.51	4.23±0.62	4.28±0.52	4.27±0.54
Postop^††^ Sodium (mmol/l)	132.27±24.61	136.91±11.71	138.05±3.74	135.93±15.38	136.20±14.42
Postop^††^ Potassium (mmol/l)	3.63±0.40	3.86±0.49	3.60±0.41_ _^*∗*^	3.86±0.48	3.83±0.48
Pain					
Preop^†^ Pain (VAS)	2.12±1.56	1.86±1.62	1.86±1.21	1.91±1.66	1.90±1.61
Postop^††^ Pain (VAS)	1.46±2.11_ _^*∗*^	0.85±1.43	1.86±2.15_ _^*∗*^	0.81±1.41_ _^*∗*^	0.94±1.56

^*∗*^P<0.05. ^*∗∗*^Diagnosed with Diagnostic and Statistical Manual of Mental Disorders, 4^th^ ed., text revision (DSM-IV-TR). ^+^The estimated volume of blood loss was determined through Mercuriali's formula.^++^P<0.001. ^†^The sample was taken on the first or second hospital day, before operation. ^††^The sample was taken on the second postoperative day

**Table 3:** The symptoms of delirium and the estimated volumes of blood loss during operation*∗*(mean±SD, ml).

	With Symptom	Without Symptom	P value
Acute Onset	1225.26±1049.88 (N=27)	513.97±604.83 (N=146)	<0.001
Inattention	1012.86±1011.98 (N=28)	536.61±608.23 (N=145)	0.001
Disorganized Thinking	809.06±1412.34 (N=12)	603.83±666.25 (N=159)	0.354
Altered Consciousness	1058.72±831.14 (N=18)	576.60±709.25 (N=157)	0.008
Disorientation	1158.53±855.69 (N=22)	512.66±583.57 (N=146)	<0.001
Memory Impairment	793.69±677.68 (N=13)	609.16±743.20 (N=160)	0.388
Perceptual Disturbance	1272.04±896.40 (N=12)	570.84±702.36 (N=161)	0.001
Psychomotor Agitation	1140.82±1126.28 (N=19)	563.51±650.03 (N=156)	0.001
Psychomotor Retardation	764.45±551.60 (N=7)	623.54±747.99 (N=165)	0.623
Altered Circadian Rhythm	1068.26±855.72 (N=29)	500.60±566.31 (N=137)	<0.001

*∗*: the estimated volume of blood loss was estimated through Mercuriali's formula.

## Data Availability

The data used to support the findings of this study are available from the corresponding author upon request.
