# Corrigendum to “The Motoric Types of Delirium and Estimated Blood Loss during Perioperative Period in Orthopedic Elderly Patients”

**DOI:** 10.1155/2020/8753817

**Published:** 2020-05-09

**Authors:** Narei Hong, Jae-Yong Park

**Affiliations:** ^1^Department of Psychiatry, Hallym University Sacred Heart Hospital College of Medicine, Hallym University, Anyang-si, Republic of Korea; ^2^Department of Orthopaedic Surgery, Hallym University Sacred Heart Hospital College of Medicine, Hallym University, Anyang-si, Republic of Korea

In the article titled “The Motoric Types of Delirium and Estimated Blood Loss during Perioperative Period in Orthopedic Elderly Patients” [1], information was omitted from the acknowledgments section in error. The acknowledgments section is shown below.
